# Electrochemical properties of a lithium-impregnated metal foam anode (LIMFA FeCrAl) for molten salt thermal batteries

**DOI:** 10.1038/s41598-022-08631-0

**Published:** 2022-03-16

**Authors:** Yusong Choi, Tae-Young Ahn, Sang-Hyeon Ha, Jae-In Lee, Jang-Hyeon Cho

**Affiliations:** Agency for Defense Development, Yuseong, P.O. Box 35, Daejeon, 34186 Republic of Korea

**Keywords:** Materials for energy and catalysis, Energy storage

## Abstract

Although numerous cathode materials with excellent properties have been developed for use in molten salt thermal batteries, similar progress is yet to be made with anode materials. Herein, a high-performance lithium-impregnated metal foam anode (LIMFA) is fabricated by impregnating molten lithium into a gold-coated iron–chrome–aluminium (FeCrAl) foam at 400 °C. A test cell employing the LIMFA FeCrAl anode exhibited a specific capacity of 2627 As g^−1^. For comparison, a cell with a conventional Li(Si) anode was also discharged, demonstrating a specific capacity of 982 As g^−1^. This significant improvement in performance can be attributed to the large amount (18 wt%) of lithium incorporated into the FeCrAl foam and the ability of the FeCrAl foam to absorb and immobilize molten lithium without adopting a cup system. For thermal batteries without a cup, the LIMFA FeCrAl provides the highest-reported specific capacity and a flat discharge voltage curve of molten lithium. After cell discharge, the FeCrAl foam exhibited no lithium leakage, surface damage, or structural collapse. Given these advantageous properties, in addition to its high specific capacity, LIMFA FeCrAl is expected to aid the development of thermal batteries with enhanced performance.

## Introduction

Owing to their excellent mechanical robustness, reliability, and long shelf life, molten salt thermal batteries have been widely used as the primary power sources for guided weapon systems^1–7^. Thermal batteries are activated by the melting of a solid eutectic electrolyte into a molten salt at high temperature (500 °C)^1,2^. Typically, Li(Si) (alloy) and FeS_2_ (pyrite) are used as anode and cathode materials, respectively, in thermal batteries^3–5^. There are many reports describing thermal battery cathodes with high specific energies and high power densities using Ni-Mo-S^8^ as well as cathode materials (CoS_2_, NiS_2_, MnO_2_, etc.)^1,5,6^. However, to date, there has been limited progress on thermal battery anodes relative to that of cathodes^3–8^. The best way to enhance the performance of a thermal battery anode is to use pure lithium, which has the highest theoretical specific capacity (13,900 As g^−1^, 3862 mAh g^−1^) among anode materials^9^. However, pure lithium melts at ~ 180 °C, which is below the operating temperature of thermal batteries (500 °C). To date, Li(Si) is the best known and most widely used anode material for thermal batteries^3^. The Li(Si) anode was first developed as an anode for rechargeable high-temperature batteries at Sandia National Laboratory (SNL) in the United States during the 1980s as a substitute for the Li(Al) anode. Li(Si) has many advantages over Li(Al), including a higher discharge rate and a higher open circuit voltage^3^. In the 1980s, the Catalytic Research Laboratory (CRC) in the United States developed a new anode material known as LAN (Lithium Anode). LAN uses pure lithium and fine iron powder to immobilize molten lithium using capillary effects at the thermal battery operating temperature (500 °C)^10^. However, the high fine iron powder content (~ 80 wt% of LAN) causes the specific capacity to be lower than the theoretical capacity. Despite iron powder being adopted in LAN to prevent molten lithium leakage during high-temperature discharge, a catastrophic lithium leakage is sometimes inevitable. As soon as cell discharge begins, the solid lithium (LAN) and the solid electrolyte (eutectic salt in MgO) melt simultaneously, and the liquid lithium and electrolyte leak out of the cell due to the applied stacking pressure (4 kg_f_ cm^−2^). Here, the stacking pressure refers to the pressure applied to the cell during final assembly. Therefore, the practical application of LAN as a thermal battery anode requires that it is wrapped in a stainless steel cup, which prevents molten lithium from leaking, which remains the best technology in the thermal battery field today^11^. According to our estimation, the stainless steel cup accounts for approximately 30% of the anode electrode weight, thus reducing the real specific capacity of LAN (80 wt% of Fe) is from 2781 As g^−1^ to approximately 1946 As g^−1^. In another approach, Choi et al*.* reported a lithium-impregnated metal foam anode (LIMFA) using a salt-coated Ni foam^12^. Although this material showed the highest specific capacity of 3009 As g^−1^ among reported thermal battery anodes, Choi et al*.* still applied a cup to prevent molten lithium leakage during discharge and to sustain the applied pressure under discharge^13^. Moreover, as shown in Figure S1, an abrupt voltage collapse is observed at 60 s during discharge when lithium is impregnated into the pure nickel mesh. The mesh is easily attacked by molten lithium at 500 °C, and it is known that pure nickel has extremely low poor resistance to molten lithium above 310 °C^14^.

In this study, iron–chrome–aluminium (FeCrAl) foam was used as a substrate in place of nickel foam for LIMFA production. The aim of this work was to develop a higher-energy–density lithium anode for thermal batteries and enhance the metal foam stability during high-temperature discharge in aggressive molten lithium. The fabrication process and the discharge properties of the LIMFA FeCrAl are reported below.

## Experimental

### LIMFA FeCrAl anode manufacturing (lithium impregnation into the metal foam)

**Figure 1:**
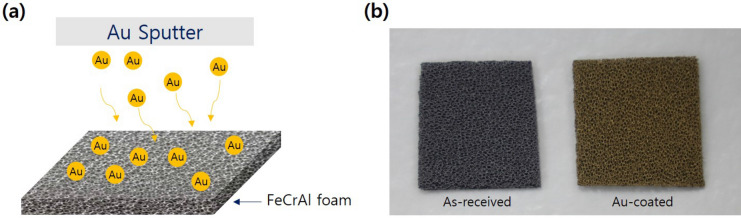
(**a**) Schematic view of Au sputtering on FeCrAl foam and (**b**) photographs of as-received and gold-coated FeCrAl foam samples.

FeCrAl foams (porosity: 90%, pore size: 800 µm, thickness: 1.8 mm; Alantum Corporation, South Korea) were used. The scanning electron microscopy (SEM) images and alloy composition of the as-received FeCrAl foam are shown in Figure S2 and Table S1, respectively. The as-received FeCrAl foam, which contained Fe, Cr, and Al components, appeared to have an embossed surface with convex features. The FeCrAl foam was cut into 40 × 40 mm pieces and then pressed (MH4389, Dong Jin Instrument, South Korea) at 1000 kg_f_ cm^−2^, which resulted in a FeCrAl foam thickness of 1.0 mm. The pressed FeCrAl foam samples were ultrasonicated in ethanol (anhydrous, 99.9% purity, Daejung, South Korea) and acetone (99.8% purity, Daejung, South Korea) for 30 min each. Then the pressed FeCrAl foam samples were plasma coated with gold (Cressington Supper Coater Q108, Cressington, UK) to modify the surface tension. The gold-coated pressed FeCrAl foam samples were impregnated with lithium in an Ar-filled glove box (KK-021AD, Korea Kiyon, South Korea) containing less than 1 ppm of H_2_O and O_2_. In a mantle furnace purged with argon (Ar, 99.999% purity, 50 sccm), lithium (50 g, 99.9% purity) was melted in a 200-mL stainless steel crucible at 400 °C^15,16^. The foam samples were immersed in the molten lithium for 1 min at 400 °C and then removed to cool to room temperature in the glove box. For comparison, as-received FeCrAl foam without gold plasma coating was also impregnated with lithium after ultrasonication in ethanol and acetone for 30 min each. Figure 1a shows a schematic view of the gold sputtering process on FeCrAl foam and Fig. 1b shows photographs of the as-received (left) and gold-coated FeCrAl foam (right). The weight of the foam was measured before and after lithium impregnation to determine the lithium content.

### Li(Si) anode manufacturing

The Li(Si) anode was prepared by mixing a Li(Si) alloy (Li content: 44 wt%, EGTECH, South Korea) with a LiCl–KCl eutectic salt (Vitzro Miltech, South Korea) in a ratio of 75:25 (w/w). After the mixture was melted at 550 °C, it was cooled to room temperature and then ground. The Li(Si) alloy/eutectic salt powder was moulded and pressed to prepare a disk-like anode pellet (outer diameter: 30 mm, inner diameter: 8 mm, pressure: 25,000 kg_f_).

### FeS_2_ cathode manufacturing

The FeS_2_ cathode was prepared by mixing FeS_2_ (> 99%, average size: 98 µm, LinYi, China), a LiCl–KCl eutectic salt (Vitzro Miltech, South Korea), and Li_2_O (> 97%, Aldrich) in a 73.5:25:1.5 (w/w/w) ratio. The addition of Li_2_O prevents the formation of a peak voltage during the initial stage of discharge. Subsequently, the mixture was moulded and pressed to produce a disk-like cathode pellet (outer diameter: 30 mm, inner diameter: 8 mm, pressure: 25,000 kg_f_) with a density of 3 g∙cm^−3^.

### Electrolyte manufacturing

The solid electrolyte was produced by melting, grinding, and pressing a LiF–LiCl–LiBr (all-Li) eutectic salt (Vitzro Miltech, South Korea) and a MgO binder (> 99%, Scora, France) in a 55:45 (w/w) ratio^17^.

### Preparation of cells

**Figure 2:**
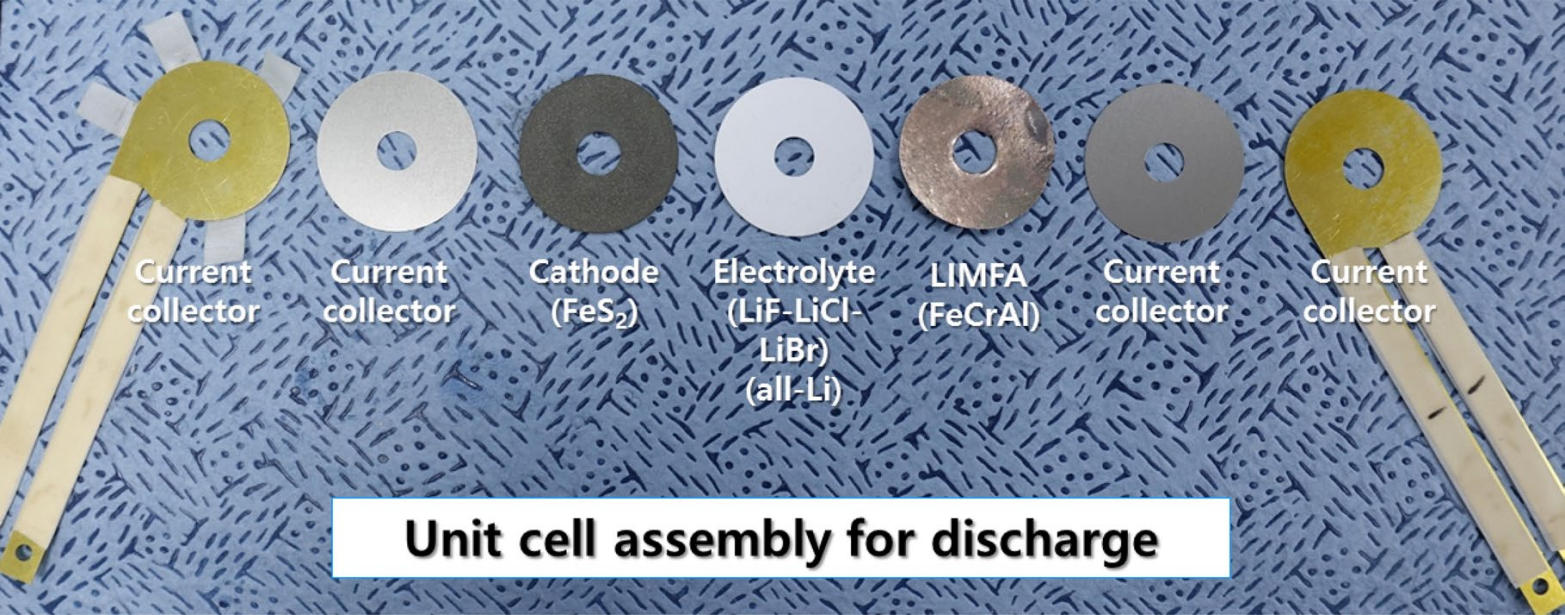
Components of a LIMFA FeCrAl unit cell. For evaluating Li(Si) unit cell discharge, LIMFA (FeCrAl) is replaced with a Li(Si) anode.

The cells (outer diameter: 30 mm, inner diameter: 8 mm) were prepared as shown in Fig. 2. The cell assembly was performed at 25 °C in a dry room with a dew point of less than − 52 °C (relative humidity < 2%). For a comparative cell discharge test with the Li(Si) anode, a cell was prepared in which the Li(Si) anode was substituted for the LIMFA anode without changing the other components. Two types of current collectors were used (steel and copper) for the electrodes and to connect the wire with electric load, respectively.

### Discharge test

The assembled cell was placed in a specially designed thermal battery cell discharge tester, which could apply heat and pressure simultaneously, similar to thermal battery operating conditions. Before introducing the assembled unit cell, the temperature of the tester was preset to 500 °C. The cells were discharged after resting for 2 min on the heated plates in the tester at 500 °C and a pressure of 4 kg_f_ cm^−2^ while applying a consecutive pulse current profile (4 A, 1 s → 2 A, 4 s → 0 A, 1 s). The cell discharge process was terminated when the voltage dropped to 0 V.

## Results and discussion

**Figure 3:**
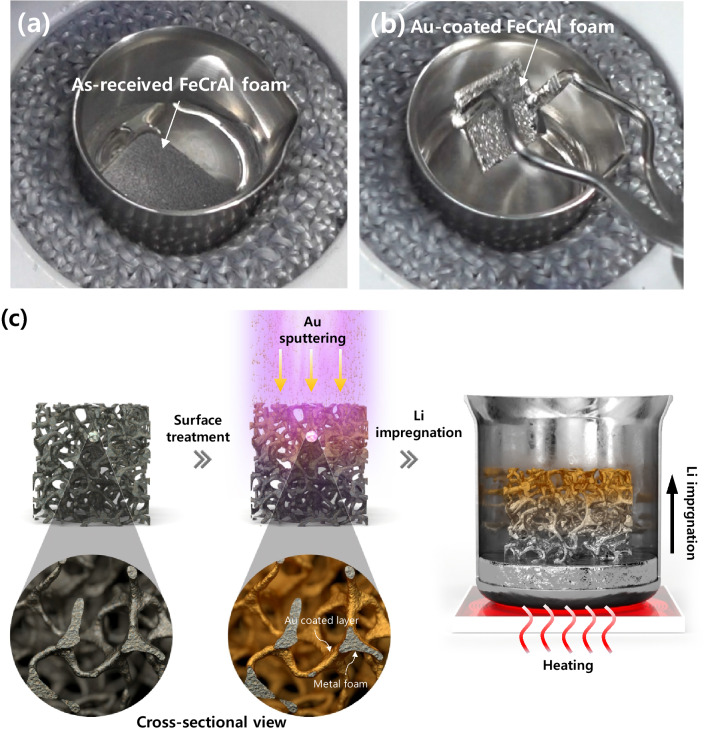
Lithium impregnation of (**a**) as-received FeCrAl foam (**b**) gold-coated FeCrAl foam, and (**c**) schematic of lithium impregnation after gold coating onto the FeCrAl foam.

The effect of gold plasma coating on the impregnation of molten lithium in the FeCrAl foam is presented in Fig. 3. As shown in Fig. 3a, lithium was insufficiently impregnated into the as-received FeCrAl foam, which is ascribed to its lithiophobic properties. In addition, even when the as-received FeCrAl foam without a gold coating was dipped into molten lithium for 40 min at 350 °C, no lithium impregnation was observed owing to the poor wettability of FeCrAl foam to molten lithium. In contrast, the gold-coated FeCrAl foam (Fig. 3b) shows good lithium impregnation, with a shiny lithium surface on the foam becoming visible within 1 min. The gold coating enhances the impregnation of molten lithium into the porous medium by modifying the surface of the FeCrAl metal foam. As shown by the cross-sectional SEM image of the gold-coated FeCrAl after lithium impregnation in Fig. 3c, Fig. S3, the impregnated lithium is evenly distributed inside the foam. Hence, the application of a gold coating on the FeCrAl foam is a useful method for improving the interfacial affinity between molten lithium and the surface of the lithiophobic FeCrAl foam. The lithium content in LIMFA FeCrAl, determined by measuring the mass of the sample before and after lithium impregnation, was 18 wt%, which is 2 wt% lower than that in the LAN (20 wt%). A major advantage is that no cup, as reported in previous research^12^, is required with the LIMFA FeCrAl to prevent molten lithium leakage during discharge. Thus, the practical specific capacity of LIMFA FeCrAl is expected to be higher than that of LAN.

**Figure 4:**
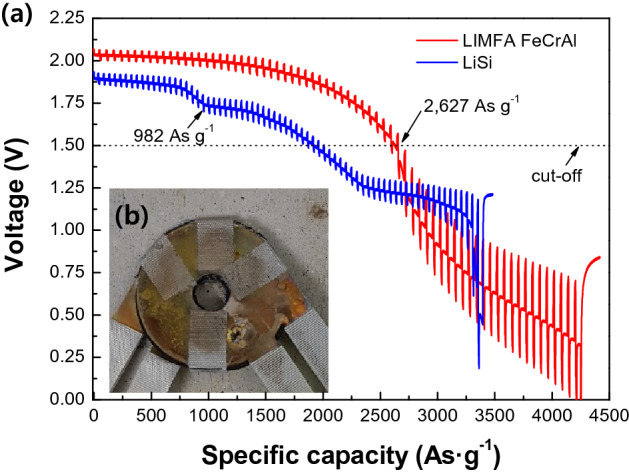
(**a**) Cell discharge performance of Li(Si) and LIMFA FeCrAl, and (**b**) image of the LIMFA FeCrAl cell after discharge.

The discharge performance of Li(Si) and LIMFA FeCrAl cells are depicted in Fig. 4a. The open circuit voltage of the LIMFA FeCrAl unit cell (2.06 V) was substantially higher than that of the Li(Si) unit cell (1.95 V), which is in accordance with a previous report^9^. These plots present the change in voltage according to the applied pulse current profile (4 A, 1 s → 2 A, 4 s → 0 A, 1 s). As a reference electrode for the anode, a FeS_2_ cathode with an almost three-fold excess electrochemical equivalent mass was intended to be used against Li(Si), as described in a previous report^10^. The fabrication of such an electrode, however, resulted in cracking during pressing. Thus, the voltage of the LIMFA FeCrAl unit cell does not present the typical cell voltage variation curve resulting from the electromotive force (emf) changes of the cathode and anode. Instead, the cell pulse discharge results exhibit three voltage plateaus that originate from the phase changes of Li(Si)^3,4^:

$${\text{Li}}_{{{13}}} {\text{Si}}_{{4}} \left( {{\text{Li}}_{{{3}.{25}}} {\text{Si}}} \right) \to {\text{Li}}_{{7}} {\text{Si}}_{{3}} \left( {{\text{Li}}_{{{2}.{33}}} {\text{Si}}} \right) \to {\text{Li}}_{{{12}}} {\text{Si}}_{{7}} \left( {{\text{Li}}_{{{1}.{71}}} {\text{Si}}} \right)$$

The voltage change of the LIMFA FeCrAl cell is solely due to lithium depletion in the FeCrAl foam. Therefore, the voltage decreases dramatically at the end of the discharge. After the full discharge of LIMFA FeCrAl, the cathode (FeS_2_) was disassembled and an SEM analysis was conducted. Interestingly, as shown in Figure S4, the cathode (FeS_2_) of the LIMFA FeCrAl cell was completely changed to Fe and Li_2_S (sulfur (S) rich area), corresponding to the end of FeS_2_ discharge in the third plateau (Fig. 4). In addition, SEM observations of the LIMFA FeCrAl after the discharge test showed that lithium was almost completely extracted and therefore is involved in the discharge reaction, as observed previously for LIMFA Ni foam^16^. After the discharge test, the appearance of the foam was similar to that before lithium impregnation. This LIMFA FeCrAl discharge behaviour needs further research. However, the third step in the voltage change of the Li(Si) cell is in accordance with the phase change of Li(Si) and FeS_2_ during discharge reported by Guidotti et al*.*^3,5^. As shown in Fig. 4, the LIMFA FeCrAl has a specific capacity of 2627 As g^−1^, whereas the Li(Si) anode shows a relatively lower specific capacity of 982 As g^−1^. The specific capacity of Li(Si) observed in this study is similar to that reported previously (1050 As g^−1^)^16^. In practice, thermal batteries commonly only use the first plateau for safety reasons, as well as strict voltage range regulations for such devices^1,2^. Consequently, the specific capacity of the LIMFA FeCrAl cell is 2.67 times higher than that of the Li(Si) cell.

**Figure 5:**
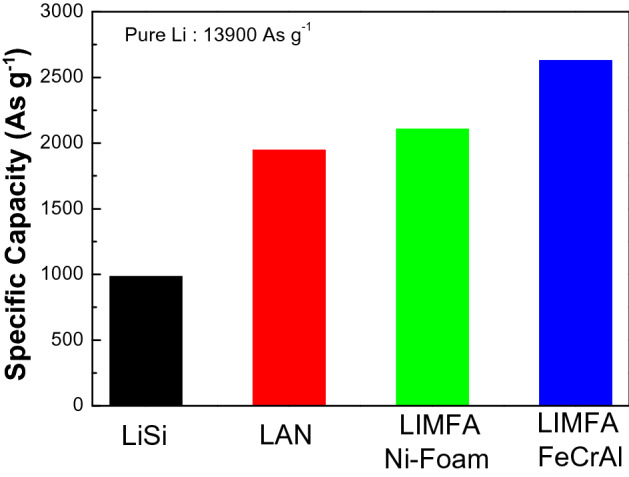
Comparison of the practical specific capacities of cells with various state-of-the-art lithium anodes with that of LIMFA FeCrAl in this study.

The specific capacity of the LIMFA FeCrAl was compared with those of various state-of-the-art lithium anodes, as shown in Fig. 5. When considering that a cup was applied, the practical specific capacity of LAN is 1946 As g^−1^ and that of the LIMFA Ni foam is 2106 As g^−1^. However, the performance of LIMFA FeCrAl is superior, with a specific capacity of 2627 As g^−1^, which, to the best of our knowledge, is the highest value that has been achieved without applying a cup. This improvement was attributed to the excellent impregnation of lithium into the high-porosity FeCrAl foam, which greatly increased the lithium content, as well as the high mechanical stability and robustness of the FeCrAl foam.

The total polarization was calculated according to the method of Fujiwara et al*.*^17,18^.

**Figure 6:**
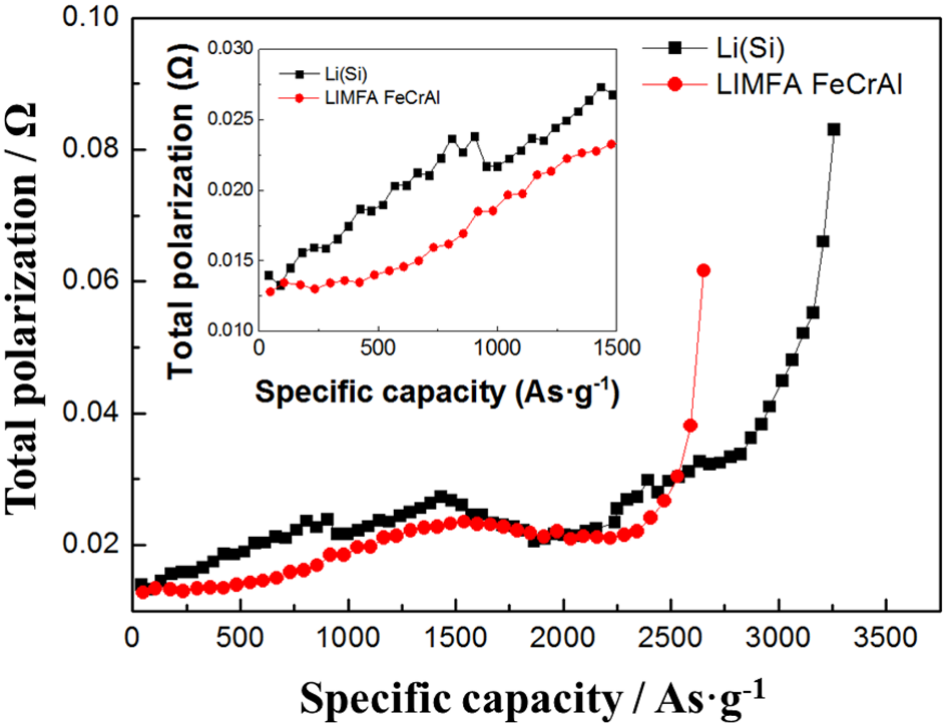
Total polarization curves of Li(Si) and LIMFA FeCrAl obtained from the cell discharge test results.

The FeCrAl foam has good mechanical robustness as well as a highly electroconductive 3D structure that can enhance ion conductivity and reduce contact resistance inside the anode. Therefore, as shown in Fig. 6, the total polarization of the LIMFA FeCrAl is reduced owing to the highly electroconductive FeCrAl foam substrate as well as the gold coating on the surface of the FeCrAl foam. The total polarization of the LIMFA FeCrAl is lower than that of Li(Si). Specifically, the total polarization of the LIMFA FeCrAl is significantly lower than Li(Si) below 1500 As g^−1^. As shown in Fig. 4, Li(Si) vs. FeS_2_ typically shows three discharge steps, but the first plateau can become dominant, as shown in Figure S5. Bernardi and Newman reported that when the lithium ratio (β) is increased to 2.51 (β = molar ratio of Li(Si)/FeS_2_) from 1.08, the % utilization of FeS_2_ in the first plateau is extended from 22.1 to 37.5% (Figure S5)^19^. In addition, Masset et al*.* reported that the conductivity of FeS_2_ decreases drastically from 80 to 100 S cm^−1^ to ~ 6.3 S cm^−1^ according to the phase change from Li_3_Fe_2_S_4_ (Z-phase) to Li_2_FeS_2_ (X-phase)^5^. The decrease in the total polarization of the LIMFA FeCrAl is attributed to the unique phase change of the extended first plateau of the LIMFA FeCrAl due to its high lithium ratio, which exceeds 2.0 (Li/FeS_2_ = 0.63 g/0.076 g). Therefore, the LIMFA FeCrAl shows higher conductivity owing to the extended first plateau of FeS_2_ and the delayed X-phase transformation of FeS_2_ in addition to the high electric conductivity of the 3D FeCrAl foam skeleton.

Without a cup on the LIMFA FeCrAl, no lithium leakage was observed and the foam frame was maintained following discharge. Therefore, the LIMFA FeCrAl developed in this study represents a significant advance for thermal battery technology.

## Conclusions

This study investigated an alternative to the conventional Li(Si) anode used in thermal batteries. The newly developed LIMFA based on a gold-coated FeCrAl foam has a higher specific capacity than the conventional Li(Si) anode. Moreover, as the FeCrAl foam has high stability and mechanical robustness in aggressive molten lithium environments, a cup for preventing lithium leakage is unnecessary. For real-world applications, the LIMFA FeCrAl has improved processability and exhibits superior performance (2627 As g^−1^) compared with Li(Si) (982 As g^−1^ at the first discharge plateau) and the iron powder method (LAN, 1946 As g^−1^). This is the first example of a thermal battery employing pure lithium without a cup. The electrochemical performance of LIMFA FeCrAl with FeS_2_ as well as the performance of this anode in a prototype stacked thermal battery need to be studied further.

## Supplementary Information

Supplementary Information.
