# Supplementary material for: Electrochemical properties of a lithium-impregnated metal foam anode (LIMFA FeCrAl) for molten salt thermal batteries
Source: Sci Rep. 2022 Mar 16;12:4474. doi: 10.1038/s41598-022-08631-0 (PMC8927342; doi:10.1038/s41598-022-08631-0)
Supplement: Supplementary file 1 — Supplementary Information. [file 41598_2022_8631_MOESM1_ESM.docx]

**Supplementary Information**

**Electrochemical properties of a lithium-impregnated metal foam anode (LIMFA FeCrAl) for molten salt thermal batteries**

Yusong Choi^a,*^, Tae-Young Ahn^a^, Sang-Hyeon Ha^a^, Jae-In Lee^a^, Jang-Hyeon Cho^a,*^

^a^Agency for Defense Development, P.O. Box 35, Yuseong, Daejeon 34186, Republic of Korea

^*^Corresponding Authors. Yusong Choi

E-mail address: richpine87@gmail.com, Tel: +82-42-821-2457; Fax: +82-42-825-3400


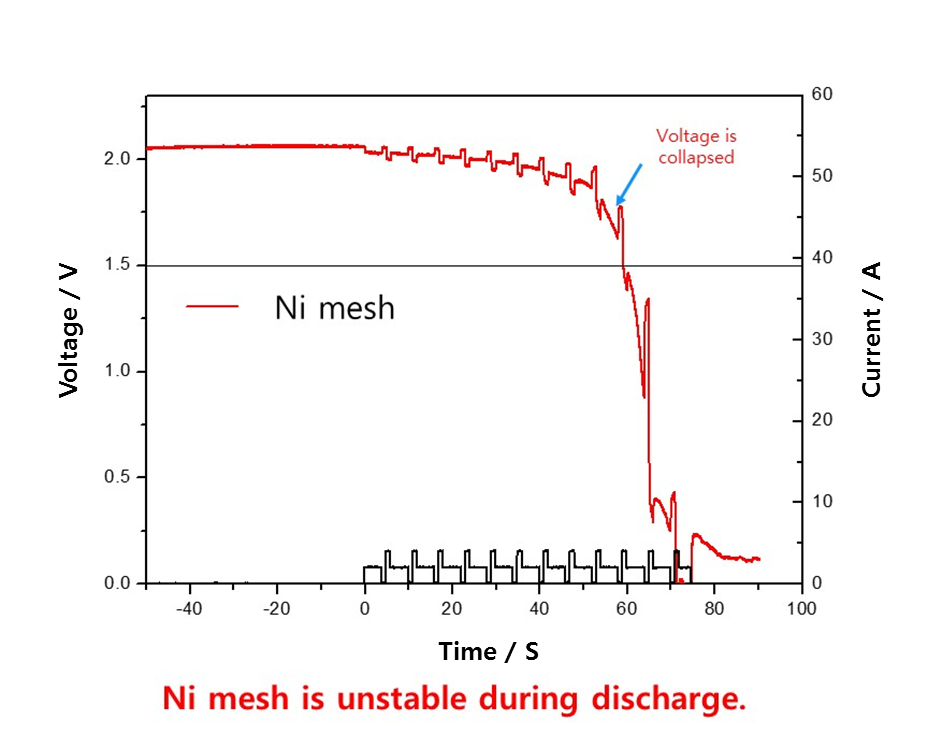


Figure S1. Unstable unit cell discharge result for lithium impregnated in pure Ni mesh owing to attack by the aggressive molten lithium. This experiment is performed with a temperature and applied pressure controlled discharger in dry room (500 ℃, 4 kg_f_·cm^-2^, Relative humidity < 3%).


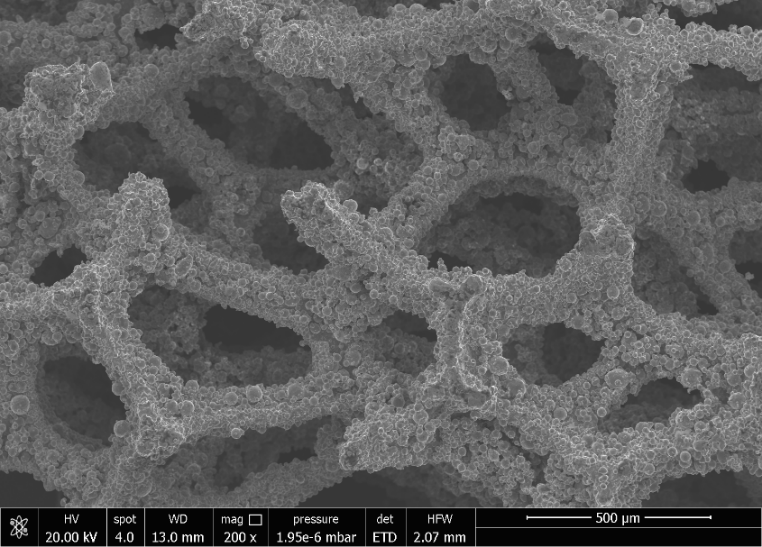


**(a)**

**(b)**


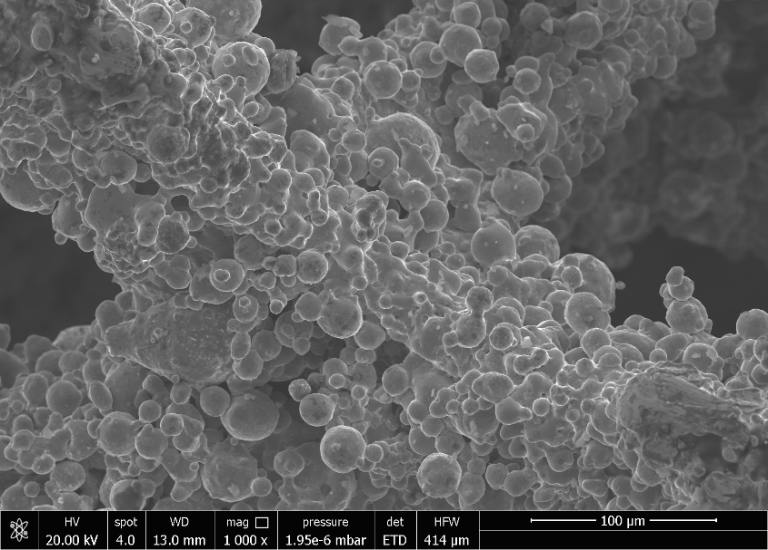


Figure S2. SEM images of as-received FeCrAl foam. (a) As-received FeCrAl foam at 200× magnification and (b) as-received FeCrAl foam at 1,000× magnification. The surface of the as-received FeCrAl foam appears embossed with convex features and Fe, Cr, and Al components.

Table S1. Energy-dispersive spectroscopy (EDS) results for as-received FeCrAl foam

| **ratio**  **element** | **wt.%** | **at.%** |
| --- | --- | --- |
| **Fe** | 66.92 | 60.88 |
| **Cr** | 25.57 | 24.98 |
| **Al** | 7.51 | 14.14 |


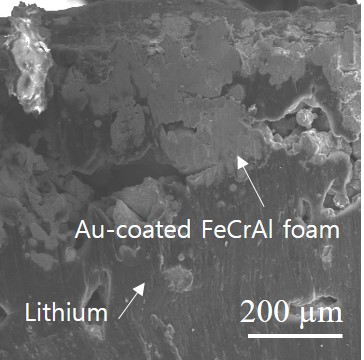


Figure S3. Cross-sectional SEM image of gold-coated FeCrAl foam after lithium impregnation (LIMFA FeCrAl).


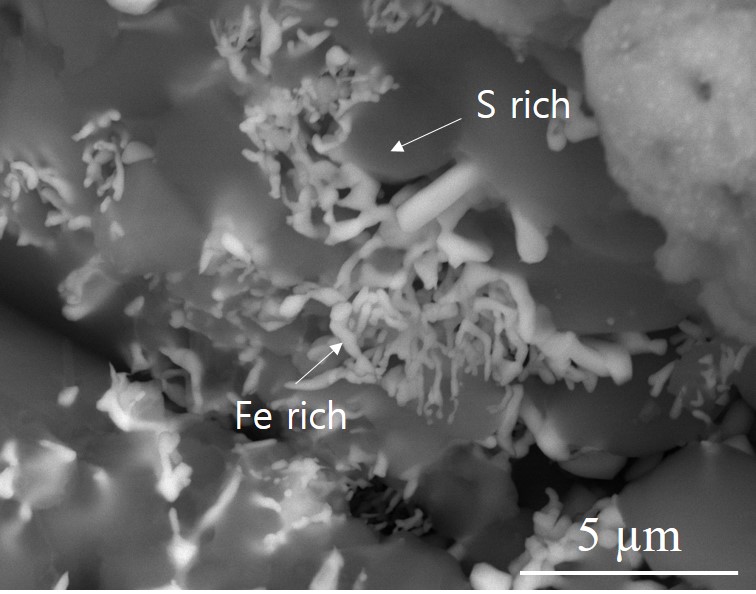


Figure S4. Cross-sectional SEM image of the cathode region after the end of full discharge of a LIMFA FeCrAl cell.


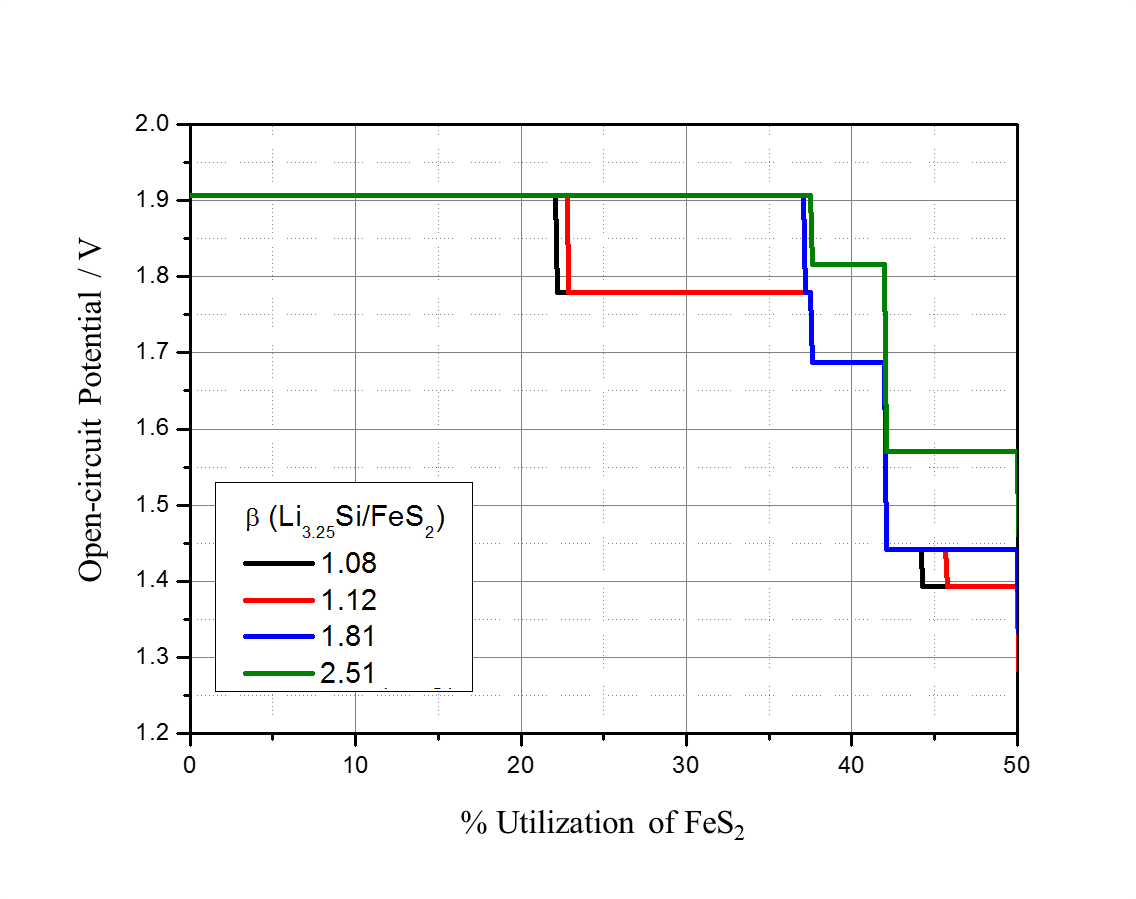


Figure S5. Open-circuit voltage estimation according to the variation in Li vs. FeS_2_ based on the method by Bernardi and Newman [1]. Where, beta (β)

Reference

[1] D. Bernardi, J. Newman, Mathematical modeling of lithium(alloy), iron disulfide cells, J. Electrochem. Soc. 134 (1987) 1309–1318.
